# Context-aware SAR image ship detection and recognition network

**DOI:** 10.3389/fnbot.2024.1293992

**Published:** 2024-01-16

**Authors:** Chao Li, Chenke Yue, Hanfu Li, Zhile Wang

**Affiliations:** ^1^School of Astronautics, Harbin Institute of Technology, Harbin, Heilongjiang, China; ^2^Key Laboratory of Space Photoelectric Detection and Perception (Nanjing University of Aeronautics and Astronautics), Ministry of Industry and Information Technology, Nanjing, Jiangsu, China; ^3^Nanjing University of Aeronautics and Astronautics, College of Astronautics, Nanjing, Jiangsu, China

**Keywords:** ship detection, synthetic aperture radar (SAR), channel-wise attention, context-aware, aggregation

## Abstract

With the development of deep learning, synthetic aperture radar (SAR) ship detection and recognition based on deep learning have gained widespread application and advancement. However, there are still challenging issues, manifesting in two primary facets: firstly, the imaging mechanism of SAR results in significant noise interference, making it difficult to separate background noise from ship target features in complex backgrounds such as ports and urban areas; secondly, the heterogeneous scales of ship target features result in the susceptibility of smaller targets to information loss, rendering them elusive to detection. In this article, we propose a context-aware one-stage ship detection network that exhibits heightened sensitivity to scale variations and robust resistance to noise interference. Then we introduce a Local feature refinement module (LFRM), which utilizes multiple receptive fields of different sizes to extract local multi-scale information, followed by a two-branch channel-wise attention approach to obtain local cross-channel interactions. To minimize the effect of a complex background on the target, we design the global context aggregation module (GCAM) to enhance the feature representation of the target and suppress the interference of noise by acquiring long-range dependencies. Finally, we validate the effectiveness of our method on three publicly available SAR ship detection datasets, SAR-Ship-Dataset, high-resolution SAR images dataset (HRSID), and SAR ship detection dataset (SSDD). The experimental results show that our method is more competitive, with AP50s of 96.3, 93.3, and 96.2% on the three publicly available datasets, respectively.

## 1 Introduction

SAR is an active microwave imaging sensor, which can obtain high-resolution radar images under low visibility weather conditions, and it is widely used in the field of ship monitoring (Yang et al., 2018), geological exploration (Ghosh et al., 2021), and climate forecasting (Mateus et al., 2012). Distinguished from other remote sensing modalities, SAR stands out due to its ability to operate day and night, under all weather conditions, and its high resolution. So it makes SAR a crucial tool for object detection and marine monitoring. Recently, scholars have shown significant interest in utilizing SAR for ship detection in ports and on the open sea, and its applications have proven vital in both military and civilian domains.

In the past decades, a series of traditional SAR ship detection methods have emerged as the research related to SAR imaging technology and surface ship detection has been continuously and vigorously developed. The most representative types of traditional methods, such as the global threshold-based method that determines a global threshold through statistical decision-making and then searches for bright spot targets in the whole SAR image (Eldhuset, 1996), adaptive threshold methods that utilize the statistical distribution of sea clutter to determine an adaptive threshold with a constant false alarm probability (Rohling, 1983) and generalized likelihood ratio methods that take into account the distributional properties of both the background clutter and the ship's target (Iervolino and Guida, 2017). However, these traditional methods are based on interpretable theoretical justifications and well-established a priori knowledge to analyze ship features in SAR images, relying on manual feature extraction. When facing complex backgrounds and SAR images with a small proportion of target pixel values, the use of manually predefined features proves challenging in extracting effective target information and eliminating background noise interference. This results in a high false negative rate in target detection, preventing the accurate identification of ship targets. With the development of convolutional neural network (CNN) and the emergence of extensive SAR image ship detection datasets, such as SAR-Ship-Dataset (Wang et al., 2019), HRSID (Wei et al., 2020), and SSDD (Li et al., 2017), which has led to the rapid development of remote sensing image-based SAR target detection techniques for ships, especially in the feature extraction of targets.

Initially, driven by a substantial quantity of publicly SAR ship datasets, several deep learning-based multi-target detectors were directly used in SAR ship detection tasks. Such as two-stage detectors, region extraction-based convolutional neural networks (RCNN; Girshick et al., 2014), FastRCNN (Girshick, 2015) and the FasterRCNN, which is representative (Ren et al., 2015). Another example is single-stage detectors such as RetinaNet (Lin et al., 2017a), SSD (Liu et al., 2016), CenterNet (Zhou et al., 2019), and YOLO series (Redmon et al., 2016; Redmon and Farhadi, 2017, 2018). The above algorithms can automatically mine the effective features of the target and no longer rely on manual extraction, but they are ineffective, those who were initially designed for use as a general-purpose object detector in visible light. Subsequently, many scholars began to consider the design of deep networks for the task of ship target detection in SAR images. For example, Ma et al. (2022) proposed a ship target detection method based on attention mechanism and key point estimation. The method uses residual link and hierarchical features to extract multi-scale targets, then uses an attention mechanism to focus on target features and detect key points to solve the dense arrangement problem. As for multi-scale problem, Zhang et al. (2022) expanded the scope of image perception region by acquiring multiple scale slices with different region sizes. In addition, they addressed the issue of false positives by calculating the distinctiveness between targets and background, and by employing a multi-ensemble reasoning mechanism to merge confidence scores from multiple bounding boxes, which enhanced the extraction of target features.

Quad-FPN (Zhang et al., 2021a) sequentially concatenated four distinct feature pyramid network (FPN; Lin et al., 2017b), progressively enhancing detection performance. Yang et al. (2021) designed the Coordinate Attention Module (CoAM), embedding positional information into channels, thereby enhancing sensitivity to spatial details and strengthening the localization of ship targets. Then, they designed the receptive field increased module (RFIM), which employs multiple parallel convolutions to construct a spatial pyramid structure, to acquire multi-scale target information.

**Figure 1:**
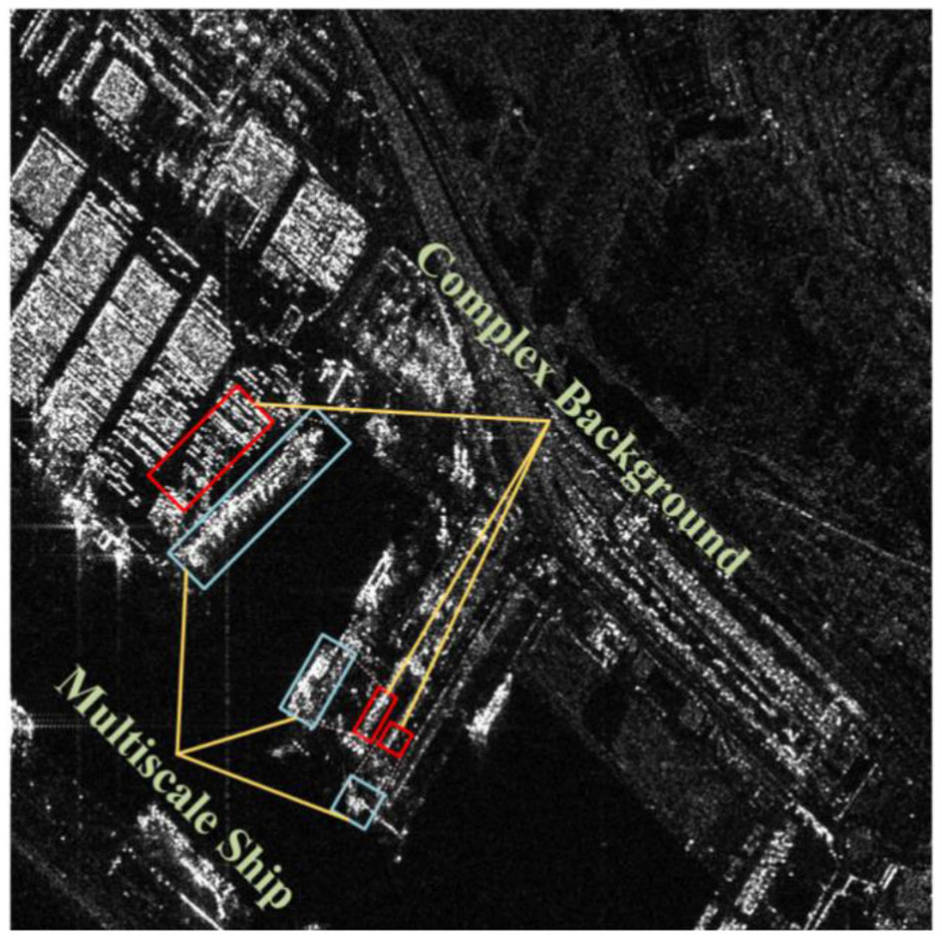
Examples of SAR images with complex backgrounds and different scales in the SAR ship dataset. The blue boxed lines show ships of different scale sizes, and the red boxed lines show the complex background noise interference that their ships may be subjected to around them.

However, in practical applications, numerous challenging issues exist, as illustrated in Figure 1. On one hand, due to the coherent imaging principles in SAR images, adjacent pixel values undergo random variations, leading to speckle noise in the image. In scenarios such as coastal ports, islands, and regions with sea clutter, SAR ship images may struggle to extract valid information, resulting in instances of both missed detections and false positives. On the other hand, the multiscaling problem poses another challenge. The varying resolutions and morphological sizes of ship targets necessitate higher demands for multiscale feature extraction from the network model, given that the pixel range occupied by ship targets can vary from a few to several hundred.

Firstly, to address the issue of significant scale variations in ship targets, we designed a LFRM, which improves upon atrous spatial pyramid pooling (ASPP; Chen et al., 2017). Apart from the first layer, a residual link is employed for each atrous convolution layer to receive and fuse the output from the previous layer, concatenating it with the current layer's output. This effectively integrates information from different scales. Finally, by combining a dual-branch channel attention mechanism using global average pooling (GAP) and global max pooling (GMP), we achieve local cross-channel interactions. The overall network architecture of our proposed method employs a multi-level design with multiple detection heads to detect targets of different sizes, making it more suitable for multiscale targets.

Secondly, to mitigate the impact of noise from a complex background on the target, we introduce the GCAM, which expands the network's sensory domain by adaptively weighting features in different spaces. It leverages estimation-based long-range dependencies to obtain global semantic features, concentrating on the target's intrinsic characteristics to weaken background noise interference. Finally, we sequentially link and embed these two modules into the Feature Pyramid Network (FPN; Lin et al., 2017b) structure with a backbone network, enabling multi-level, wide-angle perception of context. The main contributions of this paper are as follows:

- We propose a context-aware SAR image ship detection and recognition network (CANet) that effectively detects multiscale targets through both bottom-up and top-down pathways, equipped with multiple detection heads.
- A Local Feature Refinement Module (LFRM) is designed to acquire target features of varying receptive field sizes, enabling local cross-channel interactions to enhance the model's performance.
- We introduce a GCAM to capture long-range dependencies, perceive global context, strengthen target representation, and suppress noise.
- To validate the effectiveness of our approach, extensive experiments were conducted on several authoritative SAR ship detection datasets, including SAR-Ship-Dataset (Wang et al., 2019), HRSID (Wei et al., 2020), and SSDD (Li et al., 2017). Our method demonstrated outstanding performance with detection accuracies reaching 96.3, 93.3, and 96.2%, respectively.

## 2 Related work

SAR image ship target detection methods are mainly categorized into traditional methods and deep learning-based methods. The former defines ship target features manually, and then search for feature-matched ship targets in SAR images based on the predefined features, which can be categorized into three main groups: based on transform domain (Schwegmann et al., 2016), threshold-based algorithms (Renga et al., 2018) and statistical feature distribution algorithms (Wang et al., 2013). Within, the most representative one is the constant false alarm-based (CFAR-based) method. It is based on the statistical model of sea clutter, which is affected by the ocean area, the wind field conditions of the ocean, and the radar backscattering intensity varies in different wind field regions, thus forming a more complex clutter edge environment at the junction of different regions. Therefore, it is challenging to establish an accurate statistical model for a wide range of complex sea clutter. In addition, clutter modeling often requires complex mathematical theory support and time-consuming manual involvement, which also reduces the flexibility of the model and makes it difficult to effectively detect ship targets.

In recent years, convolutional neural networks (CNNS) have made great achievements in the field of natural image object detection, and their detection performance has been significantly improved compared with traditional methods. At present, natural image object detection methods based on deep learning are mainly divided into two categories: single-level object detectors and two-level object detectors. Girshick et al. (2014) proposed the first two-stage target detection model, R-CNN, which employs a traditional selective search algorithm to generate about 2,000 candidate frames, which are then fed into the CNN to extract features and categorize the candidate frames, and finally obtain the detection results. Subsequently, inspired by SPPNet (He et al., 2015), Fast R-CNN (Girshick, 2015) was proposed to solve the problem of slow detection speed of RCNN, which extracts the ROI features on the network feature map to avoid the repeated computation of features. It improved the detection speed. They used the Fully Connected (FC) layer instead of the original SVM classifier to further improve the classification performance. Ren et al. (2015), who proposed the faster FasterRCNN, designed the RPN network to replace the traditional candidate region generation algorithm selective search (Uijlings et al., 2013), which uses the convolutional network to extract the features and generate the position of the pre-selected frame. It reduces the time burden caused by the selective search algorithm and can almost reach the standard of real-time detection. More recently, faster R-CNN (Ren et al., 2015) is still the mainstream representative of two-stage detectors, and its mature design scheme has been widely used by numerous scholars.

As more demanding real-time target detection tasks are proposed, single-stage target detection is developing rapidly. As the pioneers of single-level target detectors, the YOLO series (Redmon et al., 2016; Redmon and Farhadi, 2017, 2018), by directly treating the object detection problem as the regression problem of the target region position and target category prediction, can output the positions and categories of target bounding boxes using only convolutional networks, meeting the requirement of real-time detection. Subsequently, YOLOv4 (Bochkovskiy et al., 2020) and YOLOv5 were proposed to achieve a new balance between the accuracy and speed of this series of algorithms, which were applied to more detection and recognition tasks. Another improvement of YOLO, TPH-YOLO (Zhu et al., 2021), to improve the detection accuracy of tiny targets, a tiny target detection head is added based on YOLOv5, and a total of four Prediction heads can mitigate the effects of large changes in the size of the target scale. Meanwhile, it replaces some convolutional blocks with transformer encoder ones to capture global information and sufficient background semantic information. SSD (Liu et al., 2016) and RetinaNet (Lin et al., 2017a) are two other common single-stage detectors. The former directly utilizes convolutional layers to extract detection results from different feature maps. It employs prior boxes with varying scales and aspect ratios to better match the shapes of targets, distinguishing it from YOLO, which uses fully connected layers for detection. While the latter proposes a new loss function that can be used as a more efficient alternative to previous methods for dealing with class imbalance. This class imbalance problem is solved by reshaping the standard cross-entropy loss to reduce the loss assigned to well-categorized examples.

With the blooming of deep learning in the field of images, CNN-based ship detection is increasingly subject to becoming popular. Dense Attention Pyramid Network (DAPN; Cui et al., 2019) embedded a convolutional block attention module (CBAM) into each level of the pyramid structure from the bottom up to enrich the semantic information on different level scale features and amplify the significance of features. CBAM is used to fuse the features at all levels, and the adaptive selection focuses on the scale features to further strengthen the detection and recognition of multi-scale targets. Also improved based on FPN (Lin et al., 2017b), Zhao et al. presented a novel network called attention receptive pyramid network (ARPN; Zhao et al., 2020), by fine-tuning the pyramid structure, to generate candidate boxes at different levels of the pyramid. Then, asymmetric convolution and atrous convolution are used to obtain convolution features in different directions to enhance the global context features of the local region. Then channel attention and space attention are combined to re-weight the extracted features, improving the significance of the target features and suppressing the interference of noise, and finally connect them to each layer of the pyramid laterally. Chaudhary et al. (2021) tried to directly apply YOLOv3 (Redmon and Farhadi, 2018) to ship detection and achieved some good results. Inspired by YOLO, Zhang and Zhang (2019) divided the original image into grid regions, and each grid was independently responsible for detecting the target in the region. Then, the image features are extracted through the backbone network for detection. In particular, backbone networks use separable convolution to reduce network burden.

PPA-Net (Tang et al., 2023) took into consideration that the designs of attention mechanisms such as CBAM are tailored for natural images, overlooking the impact of speckle noise in SAR images on attention weight generation. The target salience information is introduced into the attention mechanism to obtain the attention weight suitable for the SAR image. First, three pooled operations of different region sizes are constructed to obtain parallel multi-scale branches, and then activation functions are used to obtain the final channel attention weights. Meanwhile, considering the mutual exclusivity between semantic and location information and avoiding simple feature cascade operations, the authors use two self-attention weights to adaptively regulate the fusion feature ratio. To enhance the practical value of SAR ship detection applications, Zhang et al. (2019) constructed a lightweight SAR ship detection network based on the depthwise separable convolution neural network (DS-CNN). They replaced traditional convolutions with DS-CNN, significantly improving detection speed with fewer parameters, making it applicable for real-time detection tasks. Similarly, to improve detection speed, Lite-yolov5 (Xu et al., 2022a) designed a lightweight stride module and pruned the model to create a lightweight detector. To ensure detection accuracy, histogram and clustering methods were applied to enhance detection performance. Additionally, there are instance segmentation methods based on SAR ships, such as the attention interaction and scale enhancement network (MAI-SE-Net; Zhang and Zhang, 2022a). This method models long-range dependencies to enhance global perception and uses feature recombination to generate high-resolution feature maps, improving the detection capabilities for small targets. Zhang and Zhang (2022b) employed a dense sampling strategy, fusing features extracted by FPN at each layer and adding contextual information to the region of interest (ROI) to enhance information gain.

To address the issue of multiscale object detection, HyperLi-Net (Zhang et al., 2020a) utilized five improved internal modules to enhance the accuracy of multiscale object detection. These modules include multiple receptive fields, dilated convolution, attention mechanisms, and a feature pyramid to extract multiscale contextual information. Xu et al. (2022b) utilized the polarimetric characteristics of SAR to enhance feature expression and fused multiscale polarimetric features to obtain scale information. Zhang and Zhang (2020) proposed a lightweight one-stage SAR ship detection method, ShipDeNet-20. Because it uses depth-separable convolution with fewer layers and parameters instead of traditional convolution, its detection speed and model size are superior to other detection methods. Meanwhile, to ensure that the detection accuracy is not lost, features of different depths are fused to enhance the contextual semantics of features, and feature maps of the same size are superimposed to improve the expression ability of features, to improve the detection accuracy. Zhu et al. (2022) used the gradient density parameter g to construct the loss function of the network in order to solve the sparse problem of ship targets unbalanced with positive and negative ship samples. To prevent positive samples from having a decisive influence on the global gradient, the weight of the gradient proportion of multiple samples is neutralized. The author also studies the effect of the imbalance of feature levels on multi-scale ship detection. In order to ensure that semantic information is not lost during multi-layer transmission, the method of horizontal link integration of multilevel features is adopted to accelerate the flow of information so that the detailed features and semantic features can achieve balance, avoiding the semantic information and detailed features caused by the loss of other resolutions only by focusing on adjacent resolution information.

To mitigate the impact of background noise on the target, the Balance Scene Learning Mechanism (BSLM; Zhang et al., 2020b) employs a generative adversarial network (GAN) to extract complex scene information from SAR. This is followed by a clustering method to differentiate between nearshore and offshore backgrounds, thus enhancing the background. Similar balancing strategies are employed in various methods (Zhang et al., 2020c, 2021b). Additionally, some approaches utilize pixel-level processing to reduce background noise. Sun et al. (2023) used superpixels to reduce the impact of noise on the target. Firstly, the image is segmented by pixel blocks of different sizes to obtain target features of different sizes and image understanding of different semantic levels. After that, the surrounding contrast feature region is dynamically selected by dividing the size of the superpixel so that the smaller superpixel can have a larger contrast region while the larger superpixel can choose the features around itself for comparison. Finally, the superpixel features at different levels are fused for detection. Previous studies focused on extracting the features of ship targets in the spatial domain, but Li et al. (2021) believed that the spatial features of ship targets could not meet the requirements of high-precision detection, so they used the frequency domain to make up for the shortcomings in the spatial domain. Like most methods, the multi-scale spatial information of the ship target space domain is obtained through hierarchical learning, and then the invariance features of the target in the frequency domain are obtained by using the Fourier transform in polar coordinates. Finally, the features in the two-dimensional domains are compactically fused to obtain the multi-dimensional representation of the target features. In order to better adapt to the differences brought by SAR images collected by different sensors, Zhao et al. (2022) proposed an adaptive learning strategy based on the adversarial domain. Considering the different polarization modes and scattering intensity of SAR images, in order to realize the alignment of instance-level objects and pixel-level features between different domains (different sensor images), the concept of entropy is introduced as a feature weight coefficient to distinguish regions with different entropy. Since the entropy of the uniform region in SAR images is lower than that of the non-uniform region, adding entropy-based adversarial domain adaptive learning to different layers of the backbone network can effectively deal with the relationship between entropy and different receptive fields so that different domains can be aligned at the feature level as much as possible. At the same time, assigning different weights to regions with different entropy can help to distinguish the alignment results better. With the aim of distinguishing different instance-level target characteristics and make better alignment, the domain alignment compensation loss is constructed. In order to extract more precise feature information so that more uniquely representative example features can be accessed, the result of the highest score in the clustering is used to calculate the weight of the class. Zhou et al. (2023) added an edge semantic branch to solve problems such as confusion in edge detection caused by overlapping targets and used convolution of deeper and larger convolution kerns to expand the learning of context edge semantics and decouple the learned rich features, which is conducive to accurate localization of ship targets and prediction of detection frames. In addition, considering that the size of the receptive field extracted by CNN is limited, it is impossible to analyze the context from a global perspective. Therefore, a transformer framework is introduced to acquire global context features by using a multi-head attention mechanism, thus enhancing the remote analysis capability and achieving better detection and recognition effects for large-scale targets.

## 3 Context-Aware Network

**Figure 2:**
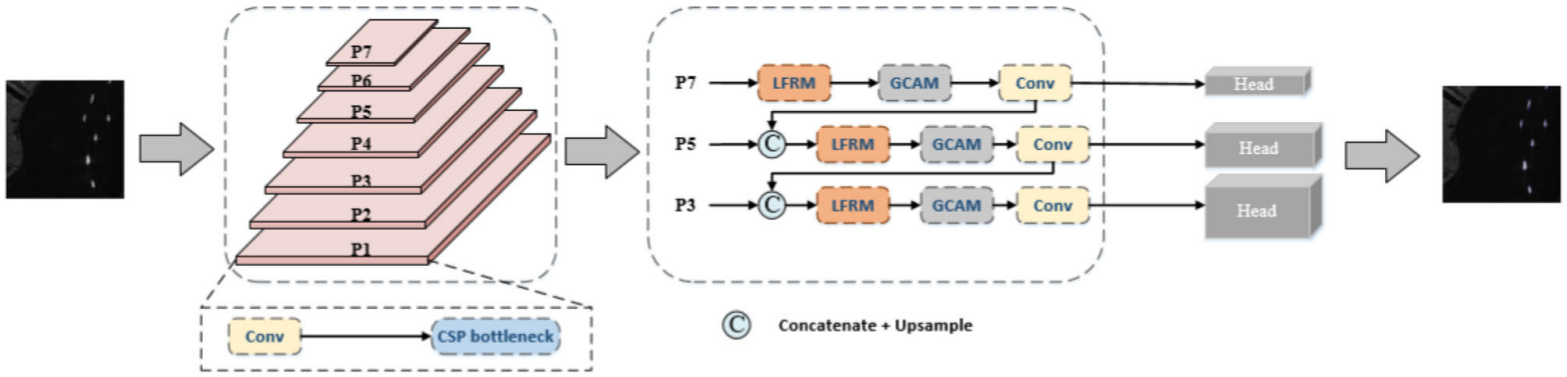
General framework of our method, where LFRM and GCAM are the proposed modules. The input image is first sent to the backbone to extract features, then passes through the FPN network structure consisting of LFRM and GCAM in series, and finally the detection results are output through the header. Where BCE loss is used for classification and objectivity and GIoU loss is used for regression.

In this section, we detail the overall architecture of the network and some other design-specific concepts and corresponding examples. The overall architecture of our approach is shown in Figure 2. Specifically, features are first extracted initially using CSPDarkNet53 as the backbone network. For the backbone network, our input goes through two convolutional layers to downsample the data to 1/4 of the input, where the activation function used in the convolutional layer is chosen to be the SiLU function. The SiLU function has a smoother curve as it approaches 0, controlling the output structure between 0 and 1 and achieving better results than ReLU in some applications. Then, the feature extraction method of YOLOv5 was adopted to obtain three effective feature layers with different resolutions and channel numbers through multiple C3 modules, and the three feature layers were input into the FPN network structure composed of LFRM and GCAM in series in parallel. The C3 module consists of three standard convolutional layers as well as multiple CSP Bottlenecks. The CSP Bottleneck mainly uses a residual structure, with one 1X1 convolution and one 3X3 convolution in the trunk, after which the residuals are left untouched and the inputs and outputs of the trunk are directly combined. The C3 module uses the CSPNet (Wang C. Y. et al., 2020) network structure, which still employs the residuals.

We capture multi-scale features through LFRM to better adapt to different scales of ship object information, thus obtaining a more representative feature map. Then, the long-range dependencies are captured by GCAM to enhance the feature representation of the target and suppress the interference of noise. The following subsections present detailed information.

### 3.1 LFRM

**Figure 3:**
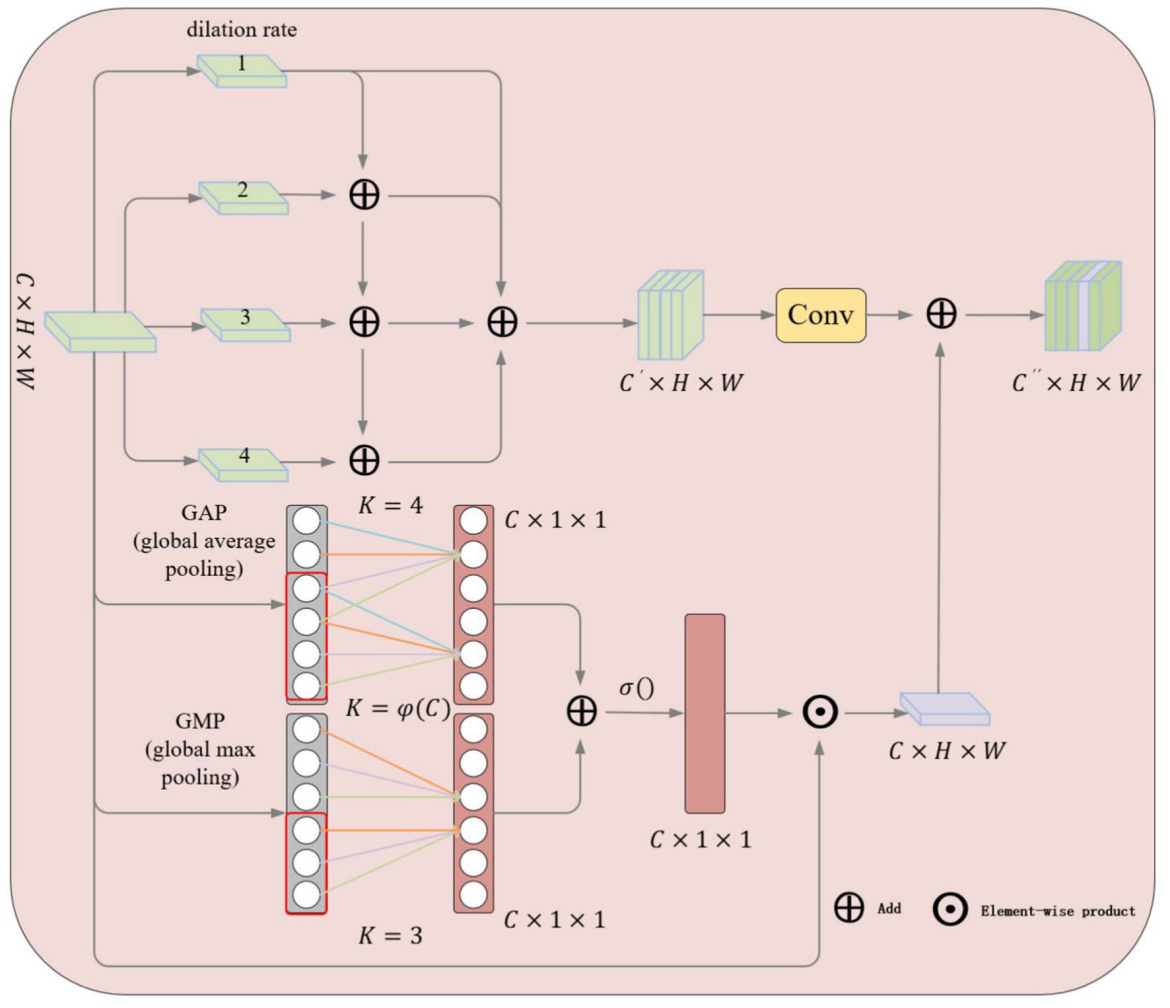
Illustration of the proposed LFRM. The upper half shows the extraction of multi-scale features using atrous convolution and the lower half shows the two-branch pooling channel attention mechanism.

Since ship targets in SAR images in real applications may have different scales, some ships may be very large while others may be relatively small, making the detection process complicated. To address this problem, we designed the LFRM module as shown in Figure 3. The deep features *x* = {*x*_1_……*x*_*i*_} obtained from the backbone network are computed in parallel by a 1 × 1 convolutional layer and three atrous convolutions with rates of 3, 6, and 12 to obtain convolutional features on multiple scales.


$$\begin{array}{l} b_{i}=Atrous\left(x_{i}\right)\\ c_{i}=Conv_{1x1}\left(x_{i}\right) \end{array}$$

After that, the feature maps *b*_*i*_ of each layer except the first one is sequentially fused with the feature maps *b*_*i*−1_ of the previous layer and activated by convolution to obtain new feature maps ${\bar{\overset{´}{b}}}_{i}$, which allows each layer to obtain a diversity of resceptive fields.


$$\begin{array}{l} {\overset{´}{b}}_{i}=Conv\left(b_{i}\right) \end{array}$$

To better fuse the different scales of information, the four obtained feature maps are finally superimposed in the channel dimension using the Concat operation and then fed into the convolutional layer to obtain a new multi-scale feature map *s*_*i*_.


$$\begin{array}{l} s_{i}=Conv\left(Concat\left({\overset{´}{b}}_{i},c_{i}\right)\right) \end{array}$$

For the purpose of enhancing the generalization ability of the network, we improve ECA-Net (Wang Q. et al., 2020) by learning the correlation between channels and adaptively adjusting the weights of the channels to improve the performance of the network. As shown in the lower part of Figure 3, we first perform global maximum pooling and global average pooling operations on the feature map *x*_*i*_to obtain two global feature descriptors, respectively, *m*∈ℝ^1 × 1 × *C*^, *a*∈ℝ^1 × 1 × *C*^, *C* indicates the number of channels.


$$\begin{array}{l} m=GAP\left(x\right)\\ \;a=GMP\left(x\right) \end{array}$$

The cross-channel information interaction is accomplished by two one-dimensional convolutions, respectively, and then the weight coefficients for each channel are calculated by SoftMax normalization. Where *w*_*i*_is the result of channel interactions, $w_{i}^{j}$ denotes the weights of the channel features, and *y*_*i*_ denotes the neighboring feature channels in a one-dimensional space. K is the result computed by the given formula, and *i* denotes the number of channels, *j*∈ ℝ^*K*^.


$$\begin{array}{l} \omega_{i}=\left(\overset{K}{\underset{j=1}{\sum }}w_{i}^{j}y_{i}^{j}\right),y_{i}^{j}\in \Omega_{i}^{K} \end{array}$$

Where the convolutional kernel size K is self-adapted by a function that allows layers with a larger number of channels to interact across channels more often. The adaptive convolutional kernel size is calculated as,


$$\begin{array}{l} K=|\frac{\log_{2}\left(C\right)}{\gamma }+\frac{b}{\gamma }{|}_{odd} \end{array}$$

Which γ = 2 , *b* = 1, |*t*|_*odd*_ is the nearest odd number to *t* and *C* is the number of channel.

Finally, the results of the two different pooling branches are superimposed according to the channel dimension, and the weight coefficients for each channel are obtained using SoftMax normalization, and*x*_*i*_ is attentively weighted according to the channel dimension.


$$\begin{array}{l} p=\sigma \left(Concat\left(\overset{´}{m},\overset{´}{a}\right)\right)\cdot x \end{array}$$

σ is SoftMax function, · is the element-wise product.

Finally, the multiscale feature *s* is overlaid with the feature map *p* after local cross-channel interaction to obtain the final LFRM output.

Since using only GAP to extract global features does not capture the detail information well, GM is added to enhance the grasp of details, and the two pooling branches complement each other to enhance the extraction of local semantic features.

### 3.2 GCAM

**Figure 4:**
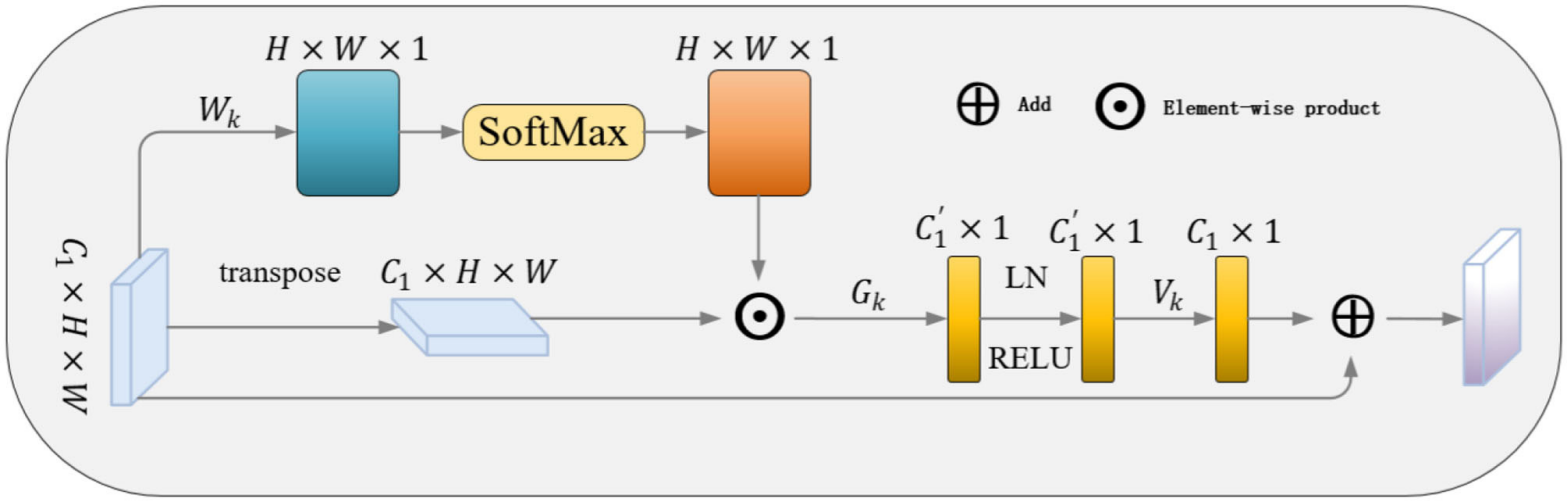
Illustration of the proposed GCAM.

To obtain remote dependent features and thus global context information to enhance the ontological target characteristics and to remove the interference of complex background noise on the target, we design the GCAM module as shown in Figure 4, where we take the multi-scale information obtained from the LFRM module as an input to obtain the remote context information about the local features.

As shown in Figure 4, it given the output of the LFRM*P* = {*P*_1_……*P*_*i*_ } as input, $P_{1}{\in \mathbb{R}}^{1\times C}$ is the feature vector at pixel *i* with *C* channel. The global context feature *f*_*i*_ is obtained by estimating the relationship between the current pixel and all pixels. After that, the weight coefficients are matrix multiplied with the local features to aggregate the contextual information (matrix multiplication is employed on the weight and local feature to aggregate contextual information).


$$\begin{array}{l} f_{i}=\overset{H\times W}{\underset{j=1}{\sum }}\frac{e^{n\left(p_{i}\right)}}{\sum_{m=1}^{H\times W}e^{n\left(p_{m}\right)}}\;*\;p_{j} \end{array}$$

Where *n*(*p*_*i*_) = *W*_*k*_*p*_*j*_ and *n*(*p*_*m*_) = *W*_*k*_*p*_*m*_ represent linear transform matrices, and *W*_*k*_ implements the 1 × 1 convolution.

With the aim of further extracting the channel dependencies while reducing the number of parameters and computational complexity, the acquisition of spatially distant effective features will be augmented by transformations, so we draw on the Non-local (Wang et al., 2018) method.


$$\begin{array}{l} {\bar{\overset{´}{f}}}_{i}=\theta \;*\;SiLU\left(\mathrm{LN}\left(\phi \cdot f_{i}\right)\right) \end{array}$$

Both ϕ and θ are realized by a 1 × 1 convolution. And the normalization (LN) and SiLU activation layers are added after the first convolution to improve the generalization of the model. Finally, the transformed feature ${\overset{´}{f}}_{i}$ is element-wise added to the multi-scale local features, yielding the GCAM output ${\tilde{f}}_{i}$ which aggregates global contextual features at each pixel.


$$\begin{array}{l} {\tilde{f}}_{i}={\overset{´}{f}}_{i}+\;p_{i} \end{array}$$

The GCAM module selectively acquires distant features for each pixel based on the correlation between spatially distant pixels, which enhances the modeling capability of feature representation and reduces background noise interference. Meanwhile, the module can be easily inserted into various network models to obtain global context information.

## 4 Experimental results and analysis

In order to fully verify the validity of our proposed methods, we test them on three authoritative public data sets and compare them with several other advanced ones. In addition, to demonstrate the effectiveness of proposed LFRM and GCAM, we design ablation experiments to evaluate the validation. Finally, we provide a comprehensive analysis of the experimental results and time complexity.

### 4.1 Training configurations and datasets

All of our experiments are conducted on a GPU workstation equipped with NVIDIA RTX 3090 with 24 GB of video memory, and the operating systems are ubuntu21.0, CUDA (10.0) and cuDNN7.0. The language and framework used to build the model are python3.7 and pytorch1.1.0, respectively. For achieving fast convergence during training, with AdamW optimizer, we set the initial learning rate to 1e-3 and employ a cosine annealing strategy to adjust. Also, to ensure experimental fairness and consistency, all the methods involved in the experiments are trained and validated under the same data benchmark. The batch setting is 16 and the maximum number of iterations is 300 to find the best model parameters.

The loss function, which used for model training, consists of classification loss, confidence loss and regression localization loss. The former two chose the classical Cross Entropy (CE), while the latter adapts Complete-IoU (CIoU) Loss.

The Cross-Entropy Loss *L*_*CE*_function expression is shown below, where *p*(*x*_*i*_) is the probability distribution of the true value, *q*(*x*_*i*_)is the probability distribution of the predicted value, and **C** denotes the total number of categories.


$$\begin{array}{l} L_{CE\;}=-\overset{C}{\underset{i=1}{\sum }}p\left(x_{i}\right)\ln\left(q\left(x_{i}\right)\right) \end{array}$$

The CIOU loss *L*_*CIOU*_function expression is shown below, where ρ^2^(*b, b*^*gt*^) represents the square of the distance between the center point of the prediction box and the center point of the real box. c represents the diagonal length of the smallest outer rectangle of the two rectangular boxes. α is the parameter used to do trade-offs, and *v* is the parameter used to measure aspect ratio consistency.


$$\begin{array}{r} CIoU=IoU-\left(\frac{\rho^{2}\left(b,b^{gt}\right)}{c^{2}}+\alpha \upsilon \right)\\ \upsilon =\frac{4}{\pi^{2}}{\left(\arctan\frac{w^{gt}}{h^{gt}}-\arctan\frac{w}{h}\right)}^{2}\\ \alpha =\frac{v}{\left(1-IoU\right)+\nu }\\ L_{CIoU}=1-CIoU \end{array}$$

The CIOU loss was chosen to normalize the coordinate scales to take advantage of the IOU and initially address the case where the IOU is zero.

To more fully evaluate the superiority of our methods, AP50 is used as the main evaluation metric, compared with currently popular methods. Specifically, PR curve is a curve drawn with precision P as the vertical coordinate and recall rate R as the horizontal coordinate. The higher the accuracy of the model, the higher the recall rate, the better the model performance, and the larger the area under the PR curve. AP50 Indicates the AP value when the IoU confidence score is 0.5. In addition, we use accuracy, recall, and F1 scores for a confidence threshold of 0.4. We also use FLOPs as an auxiliary evaluation metrics to test the efficiency of the model. The formula for calculating indicators is as follows:


$$\begin{array}{r} Precision=\frac{TP}{TP+FP}\\ Recall=\frac{TP}{TP+FN}\\ Fl=\frac{2\times Precision\times Recall}{Precision+Recall}\\ AP=\int_{0}^{1}P\left(R\right)dR \end{array}$$

### 4.2 Datasets

We evaluate our proposed methods on several public SAR ship datasets, including the SAR-Ship-Dataset (Wang et al., 2019), HRSID (Wei et al., 2020), and SSDD (Li et al., 2017) datasets. All of these datasets contain real scene images of various complex scenes ship targets of different sizes and dimensions. The SAR-Ship-Dataset (Wang et al., 2019) annotated by SAR experts, which uses 102 SAR images taken by the Gaofen-3 satellite and 108 SAR images taken by the Sentinel-1 satellite, containing 43,819 slices and 50,885 ship targets. The pixels in distance and orientation are 256. Finally, the data set is randomly divided into training set, verification set, and test set, with an image ratio of 7:2:1. HRSID (Wei et al., 2020) is a public data set used for the ship detection, semantic segmentation, and instance segmentation in high-resolution SAR images. It contains 5,604 high-resolution SAR ship images and 16,951 ship instances. The construction process draws on the COCO dataset and includes SAR images of different resolutions, polarization modes, sea states, sea areas, and ports. Its spatial resolution is 0.5–3 m. We follow the original dataset paper's delineation method. For the SSDD (Li et al., 2017) dataset is obtained by downloading publicly available SAR images from the Internet and cropping the target area into 1,160 pixels of size around 500 × 500 and manually labeling the ship target positions. We select images with image index suffixes 1 and 9 as the test set.

### 4.3 Results and analysis

#### 4.3.1 SAR-ship-dataset

As shown in Table 1, our algorithms are experimentally compared with general-purpose object detection methods including Faster R-CNN (Ren et al., 2015), RetinaNet (Lin et al., 2017a), CenterNet (Zhou et al., 2019), YOLOv4 (Bochkovskiy et al., 2020), and YOLOv5, as well as SAR-specific ship detectors DAPN (Cui et al., 2019), CoAM+RFIM (Yang et al., 2021), and PPA-Net (Tang et al., 2023) on the SAR ship dataset (Wang et al., 2019). From the Table 1, it can be observed that our method exhibits strong competitiveness. Our approaches achieve precision, recall, F1, and AP50 accuracy of 93.8, 96.1, 94.4, and 96.3%, respectively. Regarding AP50 accuracy, it outperforms the two-stage detector Faster R-CNN (Ren et al., 2015) in general object detection by 5.3%, and exceeds YOLOv4 (Bochkovskiy et al., 2020) and YOLOv5 (both are single-stage detectors) by 3.1 and 0.5%, respectively.

**Table 1:** Comparison of evaluation metrics of different methods on the SAR-SHIP dataset.

**Method**	**Precision (%)**	**Recall (%)**	**F1 (%)**	**AP_50_ (%)**
Faster R-CNN (Ren et al., 2015)	90.3	91.4	90.8	91.0
RetinaNet (Lin et al., 2017a)	84.5	93.3	88.7	93.8
CenterNet (Zhou et al., 2019)	84.6	93.5	88.8	95.0
DAPN (Cui et al., 2019)	89.9	90.7	90.3	90.6
YOLOv4 (Bochkovskiy et al., 2020)	85.7	92.7	89.1	93.2
YOLOv5	93.5	95.0	**94.9**	95.8
CoAM+RFIM (Yang et al., 2021)	93.7	95.3	94.5	96.0
PPA-Net (Tang et al., 2023)	93.5	95.5	94.7	96.1
Our	**93.8**	**96.1**	94.4	**96.3**

The best results are highlighted in bold.

In addition, in comparison with SAR ship detection method DAPN (Cui et al., 2019), which primarily focuses on the scale issue of ship targets but neglects the interference and impact of noise in small targets within complex backgrounds, resulting in an AP50 accuracy of 90.6%, significantly lower than ours and other advanced SAR ship detection methods. Our approach also outperforms another anchor-free popular algorithm, CoAM+RFIM (Yang et al., 2021), by 0.3% in the AP50 metric. Despite the consideration of noise impact and the use of attention mechanisms to reduce noise effects, the latest SAR ship detection method PPA-Net (Tang et al., 2023) falls short due to relying solely on pooling operations to address multi-scale information, leading to significant information loss.

#### 4.3.2 HRSID

The HRSID dataset exhibits a more complex image background and includes a greater number of densely packed small ship targets, posing higher challenges for algorithms and allowing for a better validation of our method's effectiveness in complex background and small target detection. As shown in Table 2, our method shows an improvement of ~0.4–15.1% compared to state-of-the-art methods, benefiting from the proposed LFRM and GCAM. LFRM first extracts local multiscale information using multiple differently-sized receptive fields and then employs a dual-branch channel attention mechanism to facilitate local cross-channel information interaction between different scale features, alleviating the detection impact of scale variations.

**Table 2:** Comparison of evaluation metrics of different methods on the HRSID dataset.

**Method**	**Precision (%)**	**Recall (%)**	**F1 (%)**	**AP_50_ (%)**
Faster R-CNN (Ren et al., 2015)	88.8	77.5	82.8	78.2
RetinaNet (Lin et al., 2017a)	69.8	83.8	76.2	82.5
CenterNet (Zhou et al., 2019)	81.8	87.4	84.5	86.3
DAPN (Cui et al., 2019)	88.9	77.6	82.9	79.8
YOLOv4 (Bochkovskiy et al., 2020)	90.6	84.0	87.2	90.1
YOLOv5	92.4	89.3	91.2	92.9
CoAM+RFIM (Yang et al., 2021)	92.7	88.1	90.3	92.7
PPA-Net (Tang et al., 2023)	93.4	89.8	92.1	92.9
Our	**93.6**	**90.4**	**92.4**	**93.3**

The best results are highlighted in bold.

Furthermore, GCAM, by capturing long-range dependencies, enhances target feature representation and suppresses noise interference, enabling effective target detection in SAR ship images with different complex backgrounds. Even when compared to the latest SAR ship detection algorithms CoAM+RFIM (Yang et al., 2021) and PPA-Net (Tang et al., 2023), our method outperforms them by 0.9, 2.3, 2.1, and 0.2% for Precision (%), Recall (%), F1 (%), and AP50 (%), respectively. Similarly, across all four detection accuracy metrics, our method surpasses other general object detection methods and achieves optimal results. In terms of AP50 (%), it outperforms Faster R-CNN (Ren et al., 2015), RetinaNet (Lin et al., 2017a), CenterNet (Zhou et al., 2019), YOLOv4 (Bochkovskiy et al., 2020), and YOLOv5 by 15.1, 10.8, 13.5, 3.2, and 0.4%, respectively.

#### 4.3.3 SSDD

As shown in Table 3, the experimental results on this dataset indicate that our method is competitive, although the Precision and Recall accuracies are slightly lower than YOLOv4 (Bochkovskiy et al., 2020), CoAM+RFIM (Yang et al., 2021), and PPA-Net (Tang et al., 2023). Furthermore, our algorithm outperforms other classical methods, including Faster R-CNN (Ren et al., 2015), RetinaNet (Lin et al., 2017a), CenterNet (Zhou et al., 2019), DAPN (Cui et al., 2019), and YOLOv5. In summary, our method achieves significant detection accuracy. Additionally, the detection results on multiple datasets validate the fine generalization capability of this method.

**Table 3:** Comparison of evaluation metrics of different methods on SSDD dataset.

**Method**	**Precision (%)**	**Recall (%)**	**F1 (%)**	**AP_50_ (%)**
Faster R-CNN (Ren et al., 2015)	90.9	87.6	89.2	88.3
RetinaNet (Lin et al., 2017a)	81.6	92.3	86.6	89.6
CenterNet (Zhou et al., 2019)	93.3	**94.5**	93.9	93.5
DAPN (Cui et al., 2019)	87.6	91.4	89.4	90.1
YOLOv4 (Bochkovskiy et al., 2020)	93.6	94.0	93.8	96.1
YOLOv5	94.0	92.4	92.7	95.3
CoAM+RFIM (Yang et al., 2021)	94.4	92.1	93.2	95.6
PPA-Net (Tang et al., 2023)	94.8	**94.5**	93.3	96.0
Our	94.2	93.9	**94.5**	**96.2**

The best results are highlighted in bold.

#### 4.3.4 Visual results

**Figure 5:**
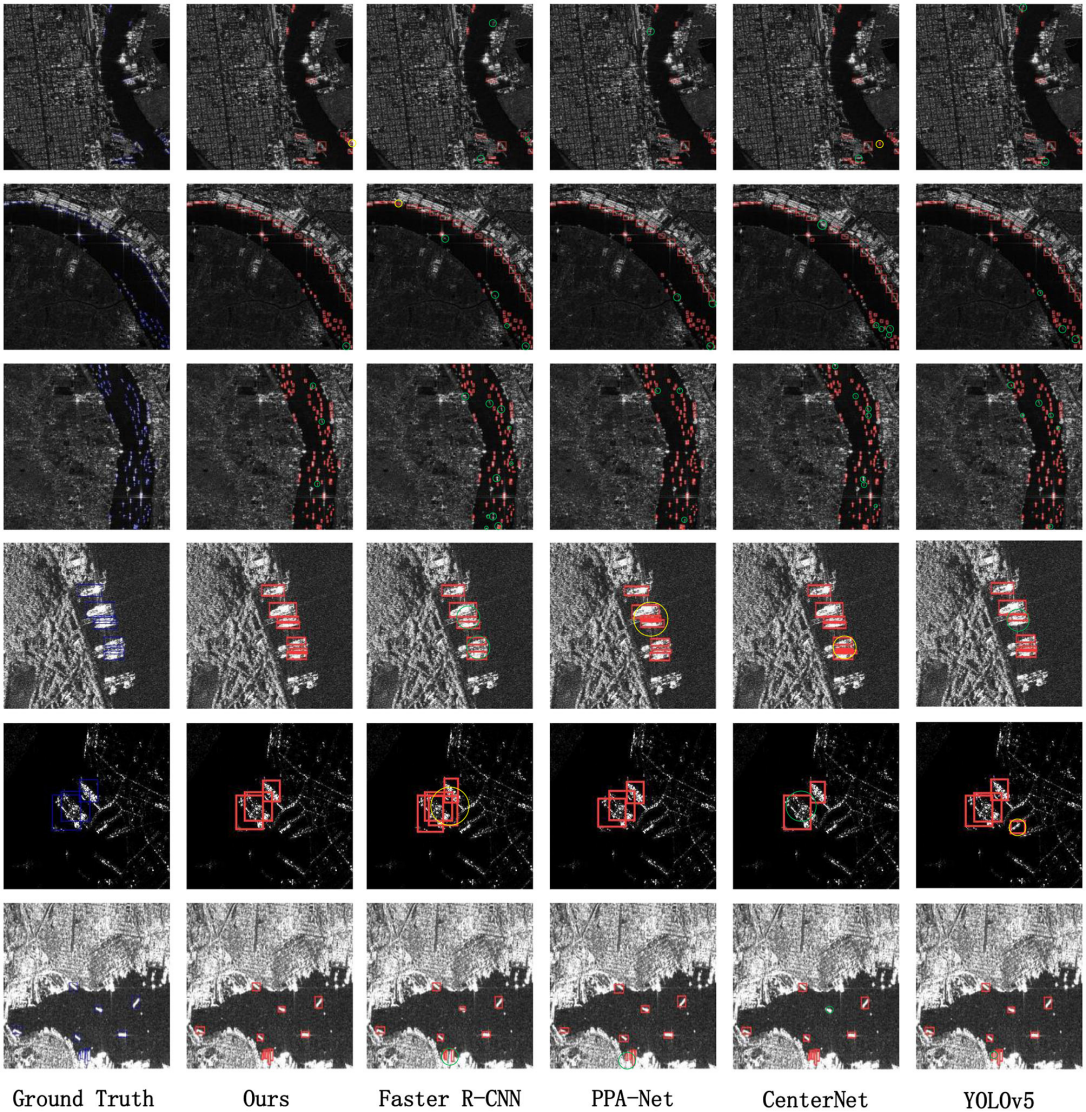
We have chosen to compare the detection results of different methods for complex backgrounds and multi-scale targets (especially small targets). The red box indicates the ground truth, and false alarms and missed detections are circled using yellow and green circles, respectively.

**Figure 6:**
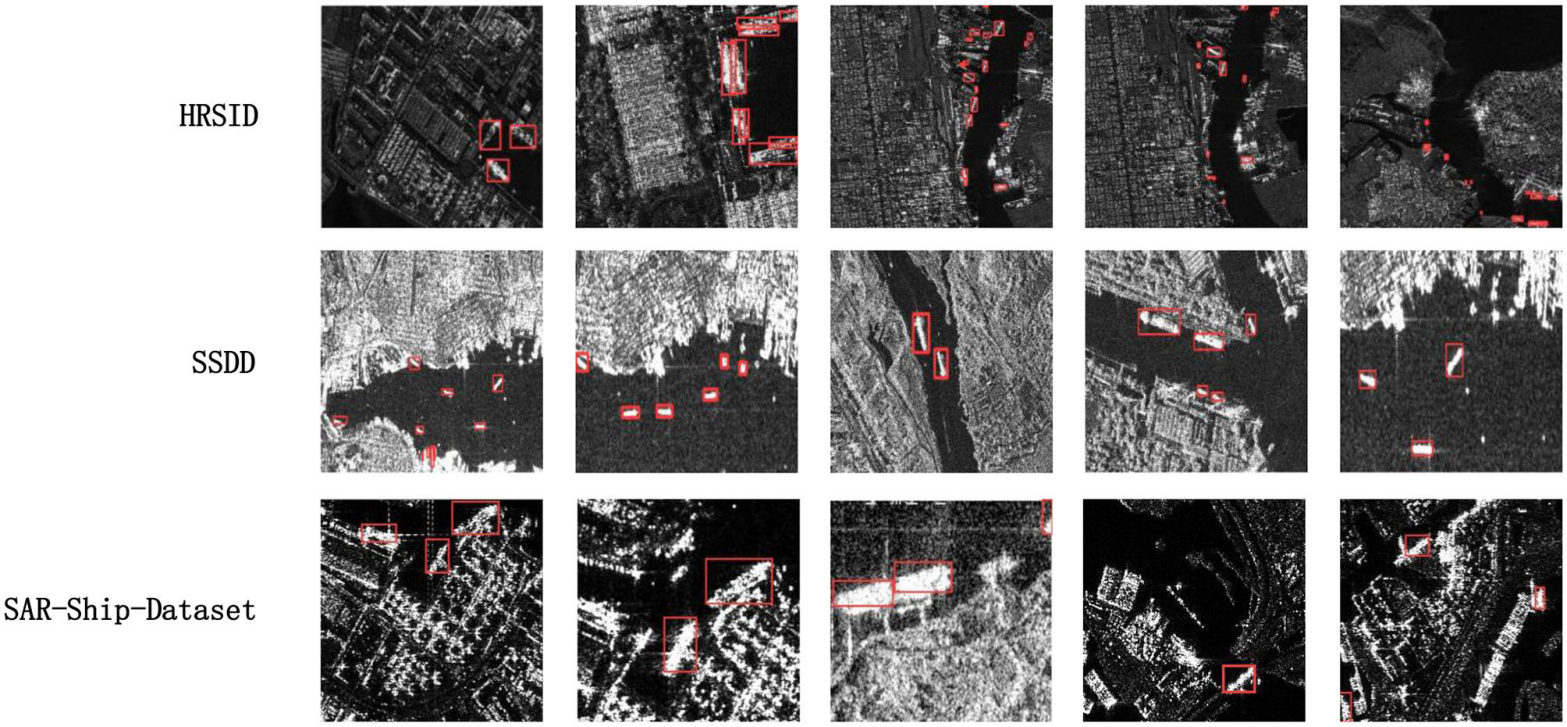
Plot of detection results for selected ships with complex backgrounds from HRSID, SSDD, and SAR-Ship-Dataset datasets for our method.

**Figure 7:**
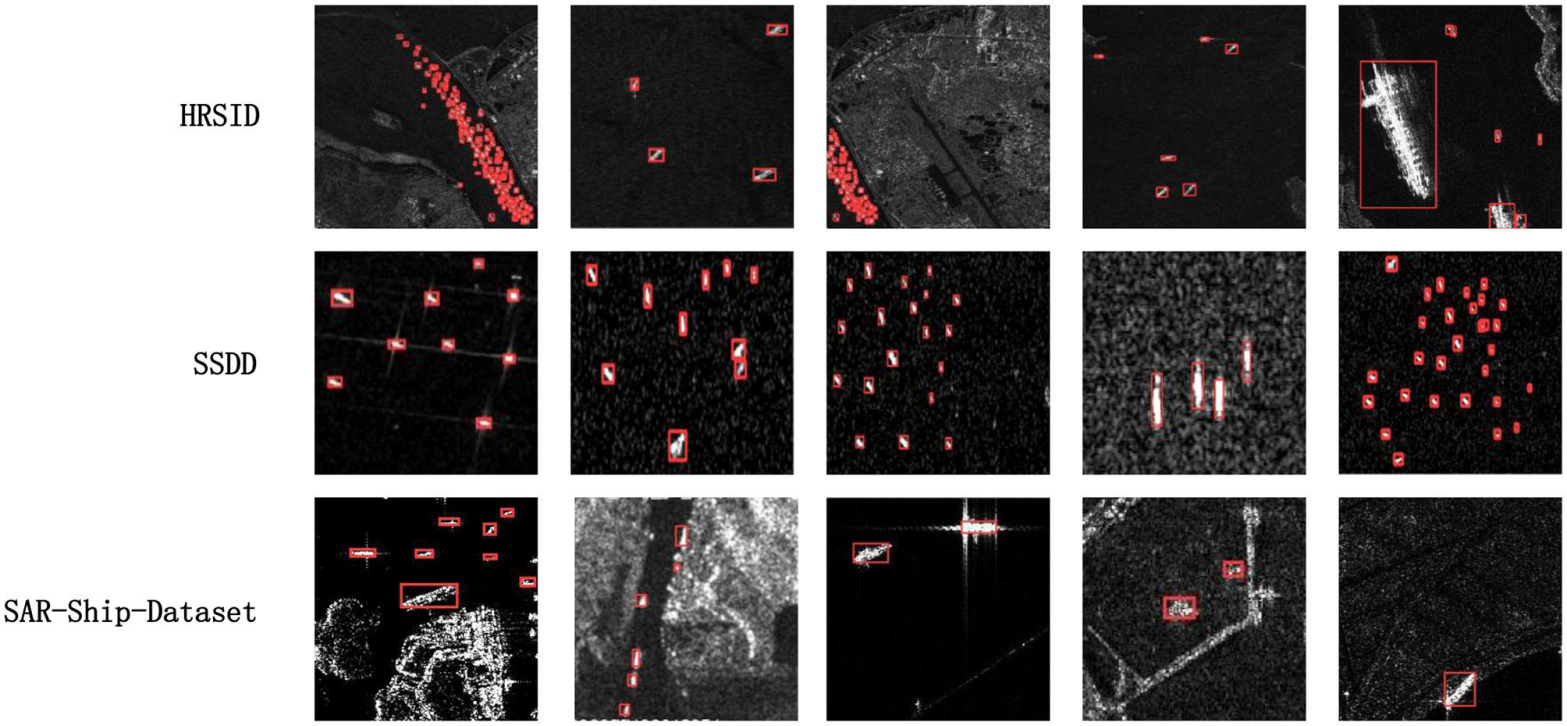
Our approach plots a selection of detection results with small targets and densely arranged ships in the HRSID, SSDD, and SAR-Ship-Dataset datasets.

To directly showcase the advanced detection results of our method, we visualize the detection outcomes on three different datasets. As illustrated in Figures 5–7, it is evident that our method performs exceptionally well in both complex background and various-sized ship targets, surpassing other approaches. Specifically, Figure 5 displays the detection results of our method and other approaches in SAR images with complex backgrounds and multiple-scale targets. It is noticeable that other methods exhibit instances of missed detections or false positives, while ours demonstrates good detection accuracy in both scenarios. Figure 6 presents the detection results of our method for ships with complex backgrounds. Figure 7 illustrates the results of detecting small target ships, consistent with our expectations that the LFRM module can effectively utilize multiple receptive fields of different sizes to extract local multiscale information, making the network more sensitive to small targets.

In summary, the visualization results intuitively reflect that our proposed method can accurately detect and identify ship targets in SAR images with complex backgrounds and various target sizes. Moreover, it demonstrates effective target detection across different datasets and diverse scenarios, offering better practical utility. However, our method exhibits some instances of missed detections and false positives in dense target detection, as shown in Figure 5, where our method displays a few missed detections in SAR images with densely packed ships, marked with green circles. This is attributed to our method solely considering the influences of multiscale targets and backgrounds, without accounting for potential feature overlap and misalignment that may arise when targets are densely arranged. Our current approach does not perform feature subdivision for overlapping targets, and we plan to address this in future work.

#### 4.3.5 Ablation study

To evaluate the effectiveness of the components in our proposed Context-Aware Network, we conduct extensive ablation experiments on the HRSID (Wei et al., 2020) dataset. For LFRM, the results are shown in Table 4, where our proposed LFRM module improves the accuracy of AP50 from 91.1 to 92.3% compared to the benchmark level. As shown in Table 5, consistent with what we envisioned, LFRM uses multi-level atrous convolution to extract feature information at different scales hierarchically, and adopts residual linking to diversify the feature receptive field at each layer, better fusing the scale features. Combined with the dual-branch channel attention mechanism to realize local cross-channel interaction, it can enhance the ability to characterize the target and efficiently filter complex semantic information. The ablation experiments also demonstrate that LFRM is not only sensitive to scale information but also can mitigate complex background noise.

**Table 4:** Ablation experiments on the HRSID dataset.

**LFRM**	**GCAM**	**AP_50_ (%)**	**Runtime (ms)**
		91.1	9.1
✓		92.3	24.3
	✓	93.0	26.9
✓	✓	**93.3**	**28.1**

We validate the effectiveness of each component step by step. It displays the AP50 (%) and the Runtime (ms). The optimal metrics have been bolded. All scores are expressed in percentage (%).

**Table 5:** Ablation experiments on the HRSID dataset for the size selection of the convolutional region K in two-branch channel attention.

**The coverage of K**	**AP_50_ (%)**	**Runtime (ms)**
3	93.0	**19.7**
4	**93.3**	20.1
5	93.1	20.4
6	92.8	20.9

The bold values indicate the best results.

**Figure 8:**
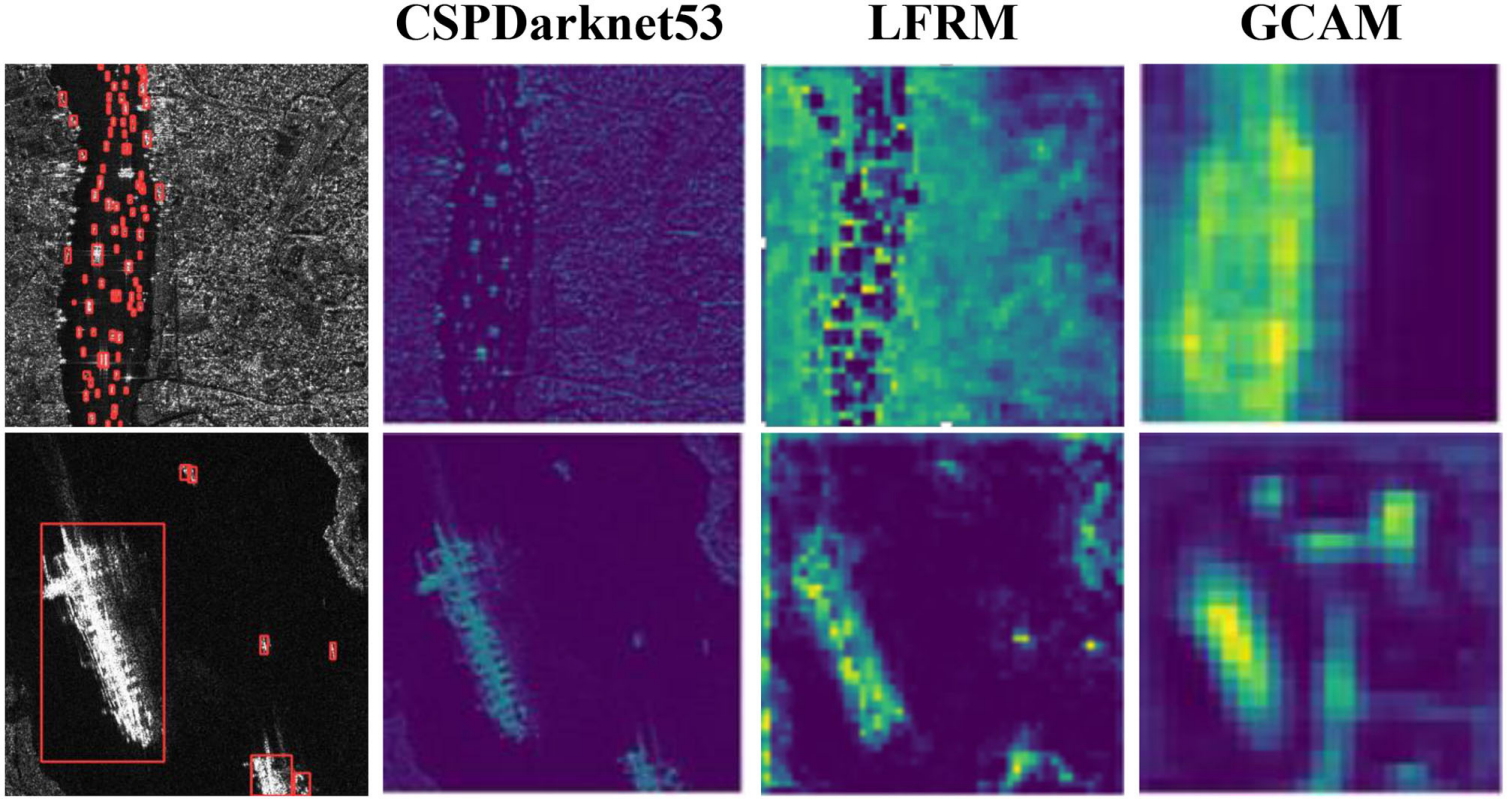
Visualization of the outputs of the different modules of the intermediate process tested by our method on the HRSID dataset.

For GCAM, our proposed GCAM module improves the accuracy of AP50 from 91.1 to 93.0% compared to the benchmark level. Essentially, GCAM expands the sensory domain of the network by adaptively weighting features in different spaces and suppresses background noise interference by obtaining global contextual information based on the estimated long-range dependency. As shown in Figure 8, to show the effectiveness of our proposed module more directly, we visualize it by outputting a visual graph of the intermediate results. Finally, by combining our two modules in series, their AP50 accuracy can reach 93.3%, which shows that the LFRM and GCAM can effectively improve the SAR ship detection performance, and the interaction can further improve our network performance.

To mitigate the impact of Batch Size on experimental results and determine the optimal Batch Size for training, we conduct ablation experiments with different Batch Size values. The experimental results are presented in Table 6. Notably, when the Batch Size reaches 16 and 32, the detection accuracy (AP50) both achieve the highest value of 93.3%. However, with a Batch Size of 8, the larger randomness introduced by the smaller Batch Size makes it challenging to converge, resulting in a lower classification accuracy of only 92.8%. When the Batch Size exceeds 32, there is a possibility of encountering local optima, leading to a decrease in accuracy to 92.9%. We exhaustively explored a range of Batch Size values in the ablation experiments to identify the most optimal Batch Size.

**Table 6:** Ablation experiments were performed on HRSID data sets with different batch sizes.

**Batch size**	**AP_50_ (%)**
8	92.8
16	**93.3**
24	93.0
32	**93.3**
36	92.9

The bold values indicate the best results.

#### 4.3.6 The complexity and speed of the network

We conduct a complexity analysis of the model, and the results are presented in Table 7. Ours has metrics of 28.1, 60.4, and 126.9 for Runtime, Params, and FLOPs, and although it is more complex to model with some other state-of-the-art methods such as YOLOv5, CoAM+RFIM (Yang et al., 2021) and PAA-Net (Tang et al., 2023), our method exhibits outstanding performance on the SAR-Ship-Dataset (Wang et al., 2019), HRSID (Wei et al., 2020), and SSDD (Li et al., 2017) datasets, delivering exceptional results while maintaining acceptable model sizes. The reason for the more complex model is that we use a more complex backbone network and GCAM in by calculating the correlation between each pixel and the other pixels, which imposes some network burden, but our method achieves a good balance for accuracy and speed.

**Table 7:** Comparison of Runtime, Params size, and FLOPs for different models.

**Method**	**Runtime (ms)**	**Params (M)**	**FLOPs (G)**
Faster R-CNN (Ren et al., 2015)	56.1	60.1	181.9
RetinaNet (Lin et al., 2017a)	55.0	55.1	175.4
CenterNet (Zhou et al., 2019)	55.0	**20.2**	63.3
DAPN (Cui et al., 2019)	74.9	63.8	266.1
YOLOv4 (Bochkovskiy et al., 2020)	22.4	64.3	110.5
YOLOv5	**19.7**	27.6	**60.3**
CoAM+RFIM (Yang et al., 2021)	37.3	65.8	123.5
PPA-Net (Tang et al., 2023)	40.2	73,9	144.5
Our	28.1	70.4	126.9

The bold values indicate the best results.

## 5 Conclusion

To address the two challenges of various complex background interferences and multi-scale ship targets in SAR image ship detection tasks, we propose a context-aware one-stage SAR ship detection algorithm. To solve the problem of multi-scale ship target detection, we propose the LFRM module, which uses dilated convolutions with different ratios to obtain multi-scale features, and then uses average and maximum global pooling to interact the extracted information of different scales, enhancing its representation ability and sensitivity to scale, and achieving multi-scale ship detection. Furthermore, we also design the GCAM module to enhance the analysis of global context information and further suppress the interference of noise from complex backgrounds on targets. Extensive experiments have demonstrated that our method outperforms the latest methods in comprehensive performance. The method proposed in this paper can effectively cope with the interference of complex background noise and detect ship targets of different scales. However, there are still some missed detection issues for densely arranged targets. In future work, we will pay more attention to the detection of densely arranged small targets.

## Data availability statement

The original contributions presented in the study are included in the article/supplementary material, further inquiries can be directed to the corresponding author.

## Author contributions

CL: Conceptualization, Writing – review & editing. CY: Writing – original draft. HL: Data curation, Writing – review & editing. ZW: Writing – review & editing.
